# Peste des petits ruminants virus (PPRV) induces ferroptosis via LONP1-mediated mitochondrial GPX4 degradation in cell culture

**DOI:** 10.1128/jvi.02310-24

**Published:** 2025-04-08

**Authors:** Qiaodi Hou, Shuijin Cheng, Zhijun Li, Congshang Lei, Yan Chen, Mingzhuo Ma, Jinming Liu, Xiwen Chen, Lizhen Wang, Qinghong Xue, Xuefeng Qi

**Affiliations:** 1 College of Veterinary Medicine, Northwest A&F University 718173, Yangling, Shaanxi, China; 2 Key Laboratory of Ruminant Disease Prevention and Control (West), Ministry of Agriculture and Rural Affairs of the People's Republic of China 12654 , Yangling, Shaanxi, China; 3 Animal Disease Prevention and Control, Mianyang Normal University 71196, Mianyang, Sichuan, China; 4 Healthy Breeding Engineering Technology Research Center, Mianyang Normal University 71196, Mianyang, Sichuan, China; 5 China Institute of Veterinary Drug Control 620909, Beijing, China; University of North Carolina at Chapel Hill, Chapel Hill, North Carolina, USA

**Keywords:** Peste des petits ruminants virus, ferroptosis, mitochondria, LONP1, innate immunity

## Abstract

Peste des petits ruminants virus (PPRV) is an important pathogen that seriously affects the productivity of small ruminants worldwide. Ferroptosis is a programmed cell death characterized by iron-dependent lipid peroxidation and the accumulation of reactive oxygen species (ROS). Emerging evidence has demonstrated that mitochondria play diverse roles in the process of ferroptosis, but the interaction between mitochondria and ferroptosis during virus infection remains largely unknown. Here, we demonstrate that PPRV induces ferroptosis, including Fe^2+^ overload, accumulation of lipid peroxidation, and shrinkage of mitochondria. Importantly, mitochondria play a crucial role in the process of PPRV-induced ferroptosis characterized by decreased mitochondrial GPX4 and lipid peroxidation in mitochondria. Mechanistically, PPRV infection downregulates mitochondrial Lon protease-1 (LONP1) expression, an important multifaceted enzyme that is essential for maintaining mitochondrial homeostasis and function, which leads to mitochondrial GPX4 degradation through the Nrf2/Keap pathway and accumulation of ROS in mitochondria. More importantly, PPRV-induced ferroptosis is tightly associated with inflammatory responses and enhanced virus replication. Overall, this study is the first to show that LONP1-mediated ferroptosis is involved in the inflammatory responses during PPRV infection.

**IMPORTANCE:**

Peste des petits ruminants virus (PPRV) infection induces a transient but severe immunosuppression in the host, which threatens both small livestock and endangered susceptible wildlife populations in many countries. Despite extensive research, it is unknown whether PPRV causes ferroptosis and what the mechanism of regulation is. Our data provide the first direct evidence that the relationship between Lon protease-1 (LONP1)-mediated dysfunctional mitochondria and the consequent induction of ferroptosis is involved in PPRV-induced pathogenesis. Importantly, we demonstrate that PPRV infection induces ferroptosis via the LONP1-mediated GPX4 degradation and ROS accumulation in mitochondria, and PPRV-induced ferroptosis is tightly associated with inflammatory responses and enhanced virus replication levels. Taken together, our research has provided new insight into understanding the effect of ferroptosis on PPRV replication and pathogenesis and revealed a potential therapeutic target for antiviral intervention.

## INTRODUCTION

Peste des petits ruminants (PPR), an acute and highly contagious disease, affects both domestic and wild small ruminants. The causative agent, PPR virus (PPRV), a linear negative-stranded RNA virus belonging to the *Morbillivirus* genus (1, 2), counteracts the type I interferon (IFN) response by multiple mechanisms (3–7) and stimulates inflammatory cytokine production (6, 8, 9). However, the exact mechanism by which PPRV regulates inflammatory responses in ferroptosis remains unclear.

Non-apoptotic regulated cell death, including ferroptosis and necrosis, leads to the release of damage-associated molecular patterns (DAMPs), which can trigger sustained inflammatory responses (10, 11). Ferroptosis is a form of regulated cell death induced by iron-dependent lipid peroxidation, regulated by various cellular metabolic and signaling pathways (12). Morphologically, ferroptotic cells exhibit ultrastructural changes in mitochondria such as volume reduction, increased bilayer membrane density, outer mitochondrial membrane disruption, and disappearance of the mitochondrial cristae (12). Mitochondria play a pivotal role in the process of ferroptosis, involving iron metabolism, the oxidative phosphorylation, reactive oxygen species (ROS), and mitochondrial membrane potential (MMP) (13, 14). Therefore, maintaining normal mitochondrial morphology and function is crucial for ensuring various cellular physiological activities (15, 16). Although the central role of mitochondria is ferroptosis, the interaction between ferroptosis and mitochondrial dynamic regulatory networks during virus infections is not yet fully understood.

Virus infections have been shown to induce cell death through diverse mechanisms, depending on the viral species. Recent findings reveal that virus can manipulate ferroptosis, promoting the proliferation, transmission, and pathogenesis (17–19). Common features of ferroptosis, such as reduced cysteine levels leading to decreased GSH and GPX4 activity, and increased cellular iron availability, have been found to occur in viral infections. Furthermore, viral infections cause extensive physiological changes in host cells, including alterations to the mitochondrial network (20, 21). While research hasn’t directly linked viral infection to mitochondrial morphology and function, evidence suggests that the loss of mitochondrial membrane integrity may disrupt many cellular processes (22). Increased focus on the mechanisms underlying ferroptosis induction during viral infections and understanding the role of mitochondria-derived factors in promoting ferroptosis may lead to discovering new therapeutic targets.

Lon protease-1 (LONP1) is a protease of the mitochondrial matrix and is a member of the highly conserved AAA^+^ superfamily. It plays diverse roles in regulating mitochondrial proteostasis and metabolism and maintaining the correct number of copies of mitochondrial DNA and cell stress responses (23, 24). As a protein quality control protease, it maintains mitochondrial morphology and function by degrading misfolded, misassembled, and oxidatively damaged proteins (25). Several studies show that LONP1 could protect against ischemia–reperfusion injury through preventing oxidative damage of proteins and lipids and maintaining mitochondrial redox balance (26). Overexpression of LONP1 remarkably improved mitochondrial morphology, thereby ameliorating liver injury and improving gluconeogenic dysfunction in acute-on-chronic liver failure (27). The mice in which LONP1 was ablated showed impaired mitochondrial ultrastructure and functions (28). These discoveries highlight the important role of LONP1 in mitochondrial and cellular homeostasis. However, the function of LONP1 in viral infections remains unclear.

In the present study, we sought to determine the involvement of ferroptosis and aberrant mitochondrial dynamics in caprine endometrial epithelial cells (EECs) upon PPRV infection. Our findings demonstrate that PPRV infection induces ferroptosis via the LONP1-mediated GPX4 degradation and ROS accumulation in mitochondria, which results in hyperactivation of inflammatory cytokines.

## RESULTS

### PPRV induces ferroptosis in EECs

**Fig 1:**
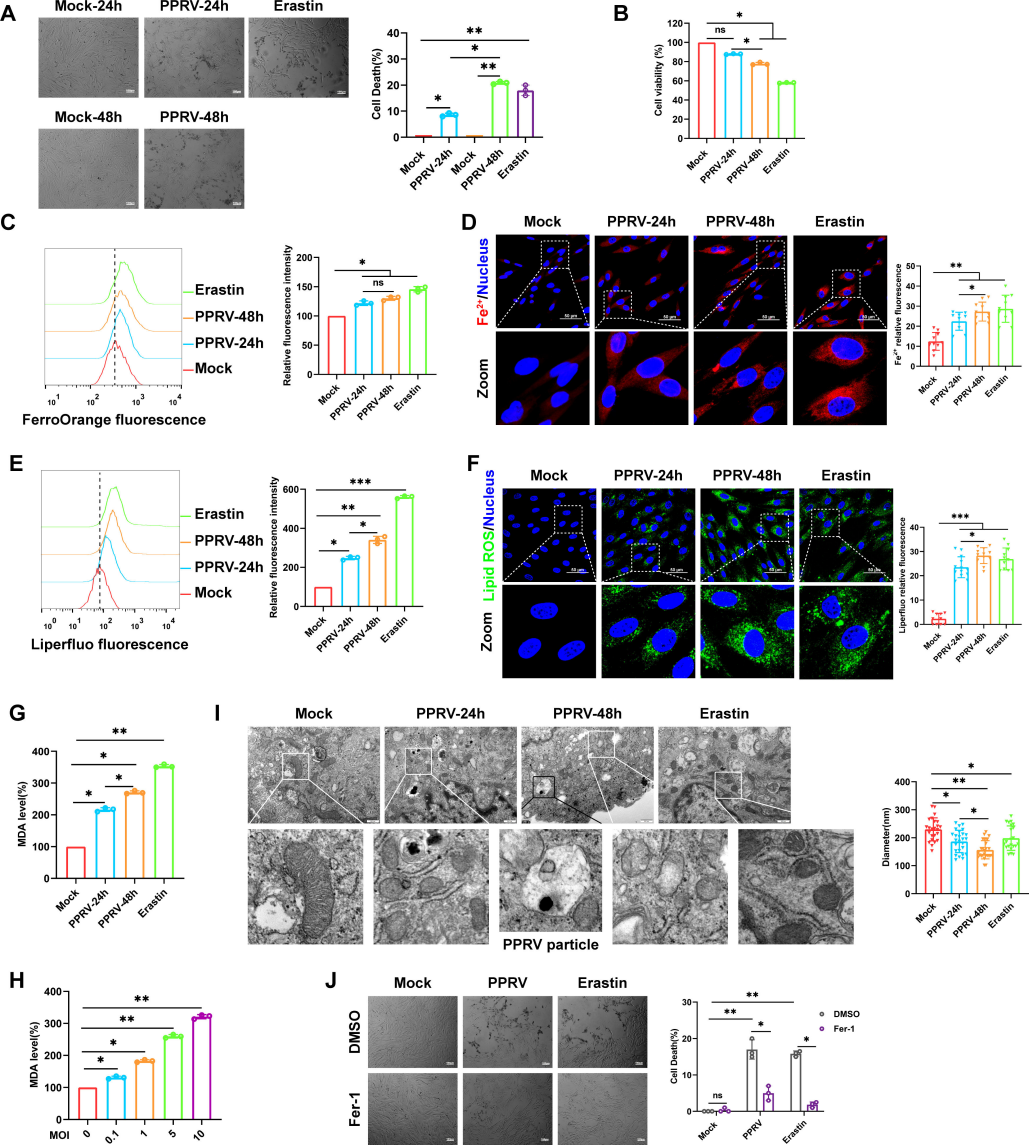
PPRV induces ferroptosis in EECs. EECs were mock-infected or infected with PPRV (MOI = 5) for 24 or 48 h, Erastin (20 µM) as a positive control. (**A**) The percentages analysis of dead cells stained with trypan blue solution, scale bar = 100µm. (**B**) Relative levels of cell viability were assayed by using CCK8. (**C**) Flow cytometry analysis of intracellular ferrous iron levels via stained with the fluorescent probe FerroOrange; the mean FerroOrange fluorescence intensity of each cell was quantified. (**D**) Representative images of fluorescence staining for ferrous iron levels in intracellular; the nucleus was stained with Hoechst 33258. Scale bars = 50 µm. (**E**) Flow cytometry analysis of intracellular lipid peroxidation levels using the fluorescent probe Liperfluo. (**F**) Representative images of fluorescence staining for lipid peroxidation levels in intracellular; the nucleus was stained with Hoechst 33258. Scale bars = 50 µm. (**G and H**) Detection of MDA concentration in cell lysates. (**I**) Representative images of normal mitochondria or dysfunctional mitochondria are shown under the TEM. Scale bar = 500 nm; the diameter of the mitochondria was quantified. (**J**) The percentages analysis of dead cells stained with trypan blue solution, scale bar = 100 µm. The data are given as mean ± SD from three independent experiments. *P* values were calculated using Student’s *t* test. An asterisk indicates a comparison with the indicated control. *P < 0.05; **P < 0.01; ***, *P* < 0.001; ns, not significant.

It has been demonstrated that ferroptosis is likely to be one of the forms of cell death during viral infections (17, 29). Here, we evaluated whether PPRV infection induces ferroptosis in EECs. Both the trypan blue staining (Fig. 1A) and cell counting kit 8 (CCK8) assays (Fig. 1B) showed that PPRV induces cell death, with the ferroptosis inducer Erastin used as a positive control (30). Since ferroptosis is mainly characterized by intracellular iron overload and redox imbalance (31, 32), we thus measured the intracellular levels of Fe^2+^ and lipid peroxidation in PPRV-infected cells using Fe^2+^ and lipid peroxidation probes, respectively. Flow cytometry and fluorescence staining analysis demonstrated that PPRV induced the accumulation of Fe^2+^ (Fig. 1C and D) and lipid peroxidation (Fig. 1E and F) at 24 and 48 h. Additionally, the levels of malondialdehyde (MDA), an indicator of lipid peroxidation (33), increased in a dose-dependent manner (Fig. 1G and H). Transmission electron microscopy (TEM) analysis revealed that, compared to mock-infected cells, the PPRV-infected and Erastin-treated cells displayed shrunk mitochondria with fewer cristae, increased mitochondrial membrane density, and reduced volume (Fig. 1I). These results further confirm the ferroptosis induced by PPRV; we next tested whether Fer-1, a potent ferroptosis inhibitor (34), could inhibit cell death in PPRV-infected cells. Compared to control cells, Fer-1 treatment rescued the cell death in PPRV-infected and Erastin-treated cells (Fig. 1J). Collectively, our data reveal that PPRV infection induces characteristics of ferroptosis in EECs.

### PPRV-induced ferroptosis enhances virus replication

**Fig 2:**
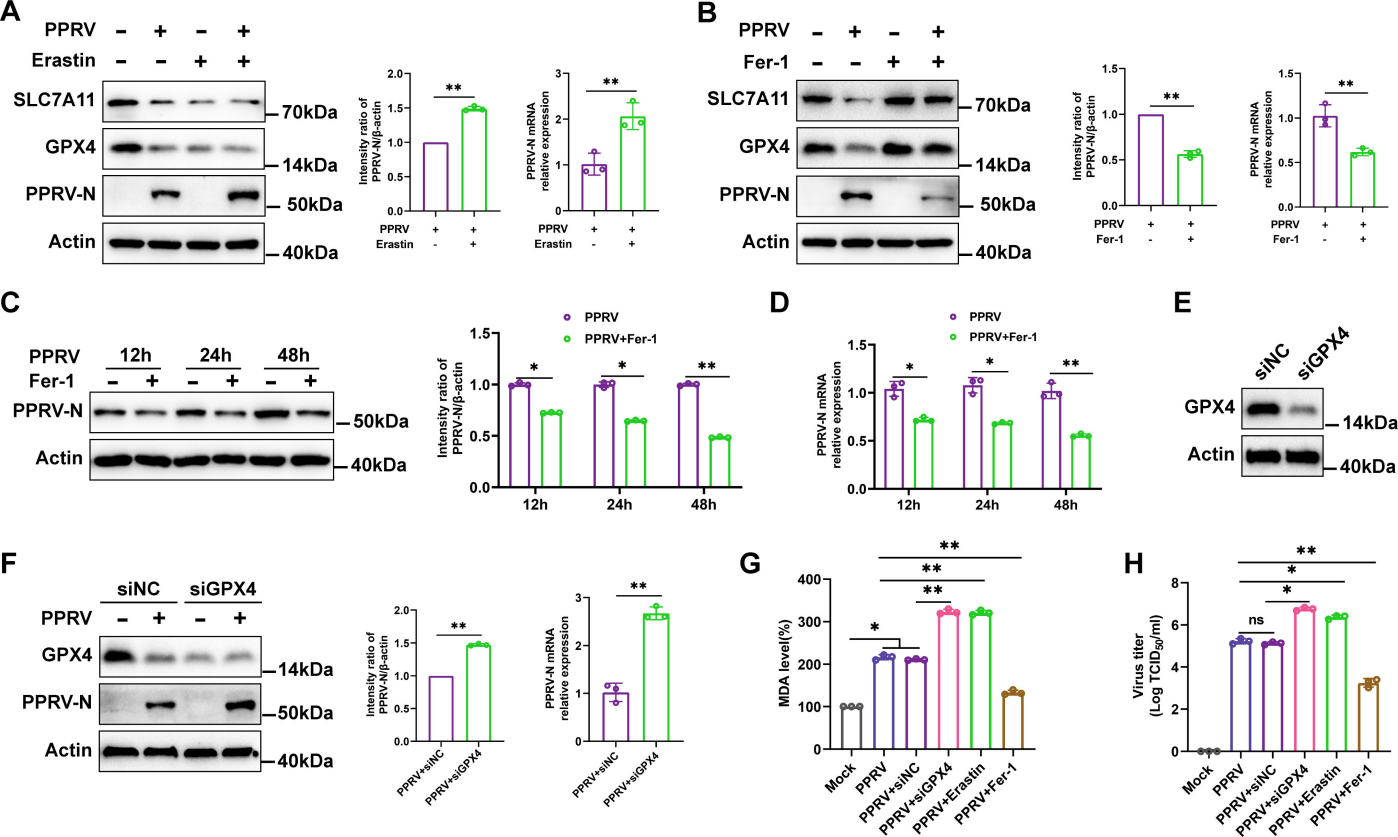
PPRV-induced ferroptosis enhances virus replication. (**A and B**) Western blotting analysis of SLC7A11, GPX4, PPRV-N, and β-actin/Actin (loading control) protein levels in EECs infected with PPRV (MOI = 5) at 48 h, with or without Erastin (20 µM) (**A**), with or without Fer-1 (20 µM) (**B** and **C**), and quantification of PPRV-N. The fluorescence intensity was quantified by ImageJ software. Real-time quantitative PCR analysis of PPRV-N (**A, B, D, and F**) mRNA levels. (**E**) Western blotting analysis of GPX4 expression in EECs transfected with siGPX4 or siNC for 48 h. (**F**) Western blotting analysis of GPX4, PPRV-N, and β-actin/Actin protein levels in mock-infected and PPRV (MOI = 5)-infected cells with or without the transfection of siGPX4 or siNC at 48 h, quantification of PPRV-N. (**G and H**) EECs were transfected with siNC and siGPX4 or treated with Erastin (20 µM) and Fer-1 (20 µM), and 48 h later, the cells were infected with PPRV at an MOI of 5; 48 h later, relative levels of intercellular MDA were assayed (**G**), and the virus titers in the supernatants were measured by TCID_50_ assay (**H**).The data are given as means ± SD from three independent experiments. *P* values were calculated using Student’s *t* test. An asterisk indicates a comparison with the indicated control. *, *P* < 0.05; **, *P* < 0.01; ns, not significant.

To investigate the effect of PPRV-induced ferroptosis on virus replication, we examined the effects of Erastin, Fer-1, and transfection of small interfering RNA (siRNA) targeting GPX4 (siGPX4) on PPRV replication levels in EECs. Our data revealed that Erastin treatment reduced the expression levels of SLC7A11 and GPX4, either in the presence or absence of PPRV(Fig. 2A ), while it increased both the mRNA and protein levels of PPRV-N gene, and virus titers in PPRV-infected cells compared to untreated cells (Fig. 2A and H). However, Fer-1 treatment abolished the decreased expression of SLC7A11 and GPX4 in the presence of PPRV(Fig. 2B). Additionally, it suppressed both the mRNA and protein levels of the PPRV-N gene (Fig. 2B) and reduced virus titers (Fig. 2H) compared to untreated cells. Furthermore, Fer-1 treatment suppressed both mRNA and protein levels of PPRV-N gene in a time-dependent manner (Fig. 2C and D). To further verify the involvement of ferroptosis in PPRV infection, we transfected cells with siRNA targeting GPX4 (siGPX4) or control (siNC), followed by PPRV infection. Western blotting analysis showed that transfection of siGPX4 effectively inhibited GPX4 expression (Fig. 2E). Knocking down GPX4 significantly enhanced both the mRNA and protein levels of PPRV-N, as well as virus titers, compared to control cells (Fig. 2F and H). Moreover, GPX4 knockdown and Erastin treatment increased MDA levels compared to respective control cells, whereas Fer-1 treatment decreased MDA levels (Fig. 2G). Together, these findings indicate that PPRV-induced ferroptosis promotes virus replication.

### Mitochondria play a key role in mediating PPRV-induced ferroptosis

**Fig 3:**
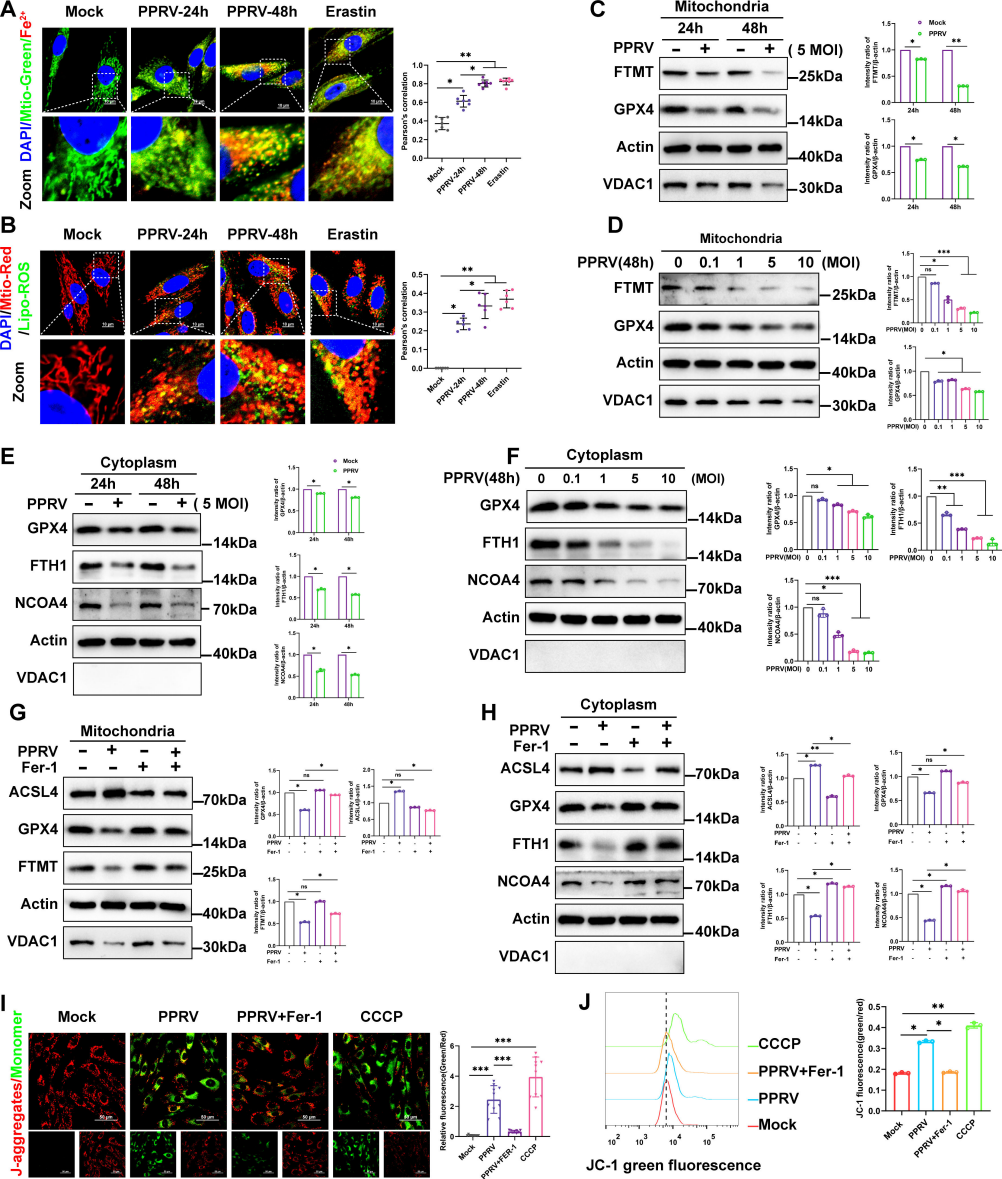
Mitochondria play a key role in mediating PPRV-induced ferroptosis. (**A and B**) EECs were mock-infected or infected with PPRV (MOI = 5) for 24 or 48 h, Erastin (20 µM) as a positive control. (**A**) Fluorescence analysis of the intracellular ferrous iron levels in mitochondria, the FerroOrange probe (red), the Mito-Tracker probe (green). (**B**) Fluorescence analysis of the mitochondrial lipid peroxidation levels, the Liperfluo probe (green), the Mito-Tracker probe (red). Scale bar = 10 µm. Colocalization analysis of fluorescence images using the colocalization plugin, which calculates Pearson’s correlation coefficient. (C to F) EECs were either mock treated or infected with PPRV (MOI = 5) at 24 and 48 h (**C and E**), or PPRV at an MOI of 0.1, 1, 5, and 10 for 48 h (**D and F**). FTMT, GPX4, β-actin/Actin, and VDAC1 protein levels in mitochondrial fractions, or GPX4, NCOA4, FTH1, β-actin/Actin, and VDAC1 protein levels in cytoplasmic fractions, were determined by Western blotting and quantification, respectively. (**G and H**) EECs infected with PPRV (MOI = 5) with or without Fer-1 at 48 h, ACSL4, GPX4, FTMT, β-actin/Actin, and VDAC1 protein levels in mitochondrial fractions (**G**) or ACSL4, GPX4, FTH1, NCOA4, β-actin/Actin, and VDAC1 protein levels in cytoplasmic fractions (**H**) were determined by Western blotting and quantification, respectively. Fractionation fidelity was verified by detection of VDAC1 in the cytosolic fraction and mitochondrial fraction. (**I**) Fluorescence analysis of MMP via JC-1 probe staining. Scale bar = 50 µm. MMP was determined by green/red ratio of mean fluorescence intensity using ImageJ software (version 1.53t) (NIH). (**J**) Flow cytometry analysis of MMP via JC-1 probe staining. MMP was determined by green/red ratio of mean fluorescence intensity. The data are given as means ± SD from three independent experiments. *P* values were calculated using Student’s *t* test. An asterisk indicates a comparison with the indicated control. *P < 0.05; **P < 0.01; ***, *P* < 0.001; ns, not significant.

Due to the pivotal role of mitochondria in oxidative metabolism (13), iron metabolism, and homeostasis (12, 35), we next explore the relationship between the induction of ferroptosis and mitochondria during PPRV infection. We monitored the accumulation of Fe^2+^ and lipid peroxidation in mitochondria using an iron probe (FerroOrange) and a lipid peroxidation probe, respectively (31, 32). Fluorescence analysis showed strong accumulation of Fe^2+^ (Fig. 3A) and lipid peroxidation (Fig. 3B) in mitochondria of PPRV-infected cells. Intriguingly, the iron and lipid peroxidation probe first appeared in a distribution that significantly colocalized with mitochondria at 24 h and subsequently also appeared in the cytoplasm at later time points (Fig. 3A and B). To explore the possible mechanisms by which PPRV-induced ferroptosis leads to excessive lipid peroxidation and Fe^2+^ accumulation, we investigated whether PPRV activates ferroptosis by inactivating GPX4, resulting in the collapse of cellular redox homeostasis (30), or by degrading iron storage protein ferritin, thereby increasing cellular iron concentration (36). First, we monitored the expression of GPX4 and the iron storage protein ferritin (FTMT and FTH) by purifying mitochondrial fractions and cytoplasmic fractions from PPRV-infected cells. Western blotting analysis revealed that PPRV infection suppressed the expression of GPX4 and FTMT in mitochondrial fractions at 24 and 48 h (Fig. 3C) and in a dose-dependent manner at 48 h (Fig. 3D). Additionally, the protein levels of GPX4, FTH1, and NCOA4 in cytoplasmic fractions were also decreased at 24 and 48 h (Fig. 3E) and in a dose-dependent manner at 48 h (Fig. 3F). No detectable expression of VDAC1 in the purified cytoplasmic fractions from both PPRV-infected and uninfected cells confirmed the absence of mitochondrial contamination in cytoplasmic fractions (Fig. 3E and F). To further verify the role of mitochondria in PPRV-induced ferroptosis, we infected cells with PPRV in the presence or absence of Fer-1. Subsequently, mitochondrial fractions and cytoplasmic fractions were purified. Our data show that treatment with Fer-1 abolished the decreased levels of GPX4 and FTMT and increased levels of ACSL4 in mitochondrial fractions from PPRV-infected cells (Fig. 3G). In addition, Fer-1 treatment also rescued the decreased levels of GPX4, FTH1, and NCOA4 and increased levels of ACSL4 in cytoplasmic fractions from PPRV-infected cells (Fig. 3H). Since MMP was used to evaluate the degree of mitochondrial dysfunction, we next analyzed whether mitochondria lost their MMP by JC-1 probe in PPRV-infected cells. We used the carbonyl cyanide 3-chlorophenylhydrazone (CCCP) as a positive control, a known inducer of mitophagy (37). Mock-infected cells exhibited normal MMP, characterized by increased red fluorescence (JC-1 aggregate) with a lesser green fluorescence (JC-1 monomer). In contrast, PPRV-infected or CCCP-treated cells exhibited decreased MMP, as shown by increased shift in red to green fluorescence (Fig. 3I). However, Fer-1 treatment rescued the MMP reduction in PPRV-infected cells (Fig. 3I). Similar results were obtained by flow cytometry analysis (Fig. 3J). Together, we conclude that PPRV infection induces changes in mitochondrial properties similar to those seen with drug-induced ferroptosis.

### PPRV regulated the GPX4-Nrf2-Keap1 pathway by downregulating LONP1

**Fig 4:**
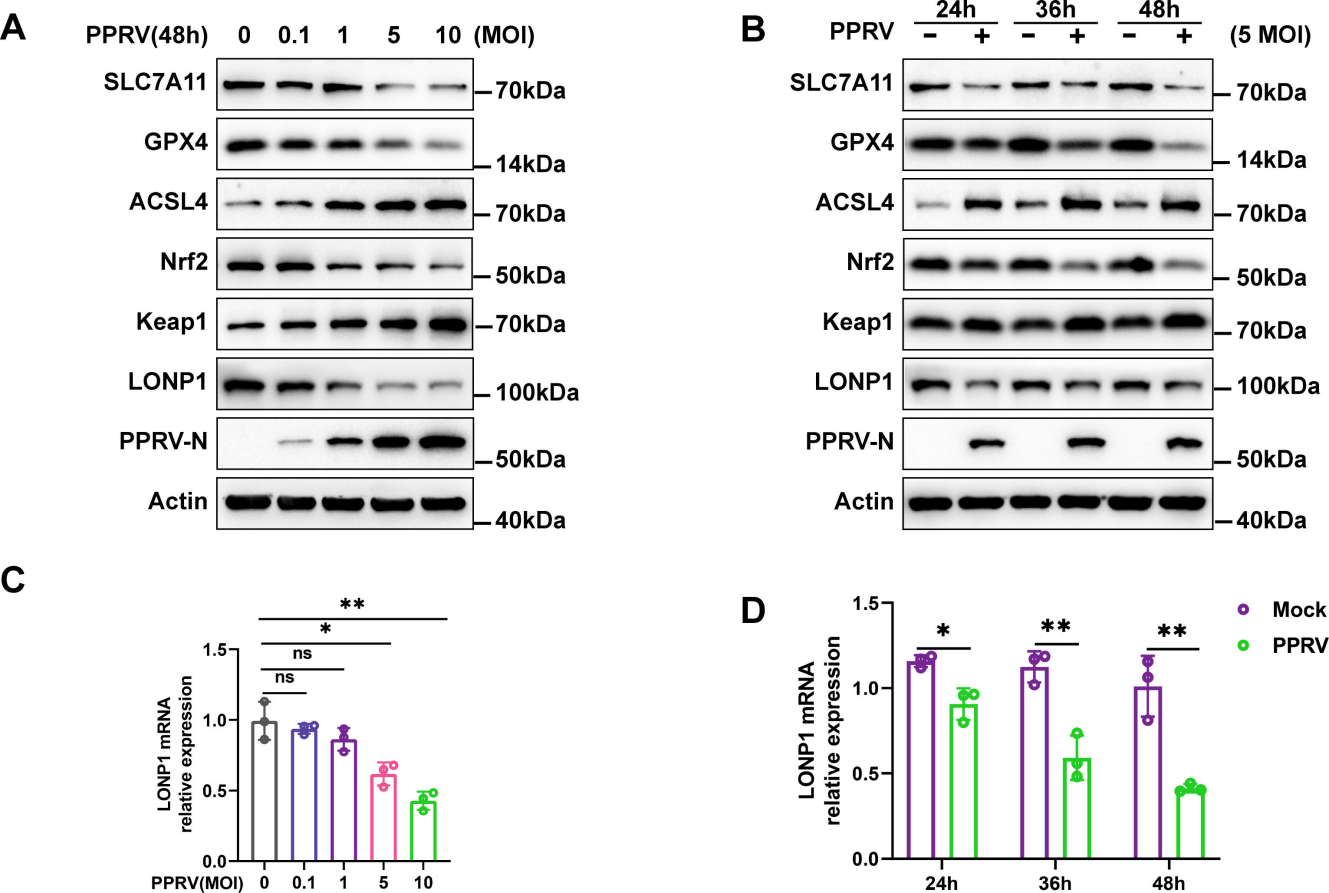
PPRV induces ferroptosis by regulating the GPX4-Nrf2-Keap1 pathway. EECs were either mock treated or infected with PPRV Nigeria 75/1 at a MOI of 0.1, 1, 5, or 10 for 48 h (**A and C**) or a MOI of 5 for 24, 36, or 48 h (**B and D**). SLC7A11, GPX4, ACSL4, Nrf2, Keap1, LONP1, and β-actin/Actin (loading control) protein levels were determined via Western blotting; PPRV-N was used as a marker for virus infection (**A and B**). Real-time quantitative PCR analysis of LONP1 genes (**C and D**). The data are given as means ± SD from three independent experiments. *P* values were calculated using Student’s *t* test. An asterisk indicates a comparison with the indicated control. *, *P* < 0.05; **, *P* < 0.01; ns, not significant.

As ferroptosis is caused by iron-dependent lipid peroxidation, the system Xc^−^/GPX4 axis suppresses lipid peroxidation and is a vital pathway involved in inhibiting ferroptosis (17, 38). Therefore, we examined whether PPRV activates ferroptosis by directly blocking system Xc^−^. Western blotting analysis revealed that PPRV infection suppressed the expression of SLC7A11 and GPX4, while increasing ACSL4 expression in EECs in a dose- and time-dependent manner (Fig. 4A and B). The results demonstrate that PPRV infection inhibits the activation of the system Xc^−^/GPX4 axis, subsequently promoting cell lipid peroxidation. Nrf2 is the key transcription factor for maintaining oxidative homeostasis and is activated under conditions of high oxidative stress, promoting target gene transcription such as GPX4, HO-1, and SLC7A11 (39). In our results, Nrf2 was also dose- and time-dependently inhibited by PPRV (Fig. 4A and B). Notably, the protein level of Keap1, the interactor and repressor of Nrf2, was increased in response to PPRV (Fig. 4A and B). Overall, our results suggest that PPRV infection inhibits activation of the system Xc^−^/GPX4 axis through the Nrf2-Keap1 pathway, which subsequently promotes cell lipid peroxidation and ferroptosis.

**Fig 5:**
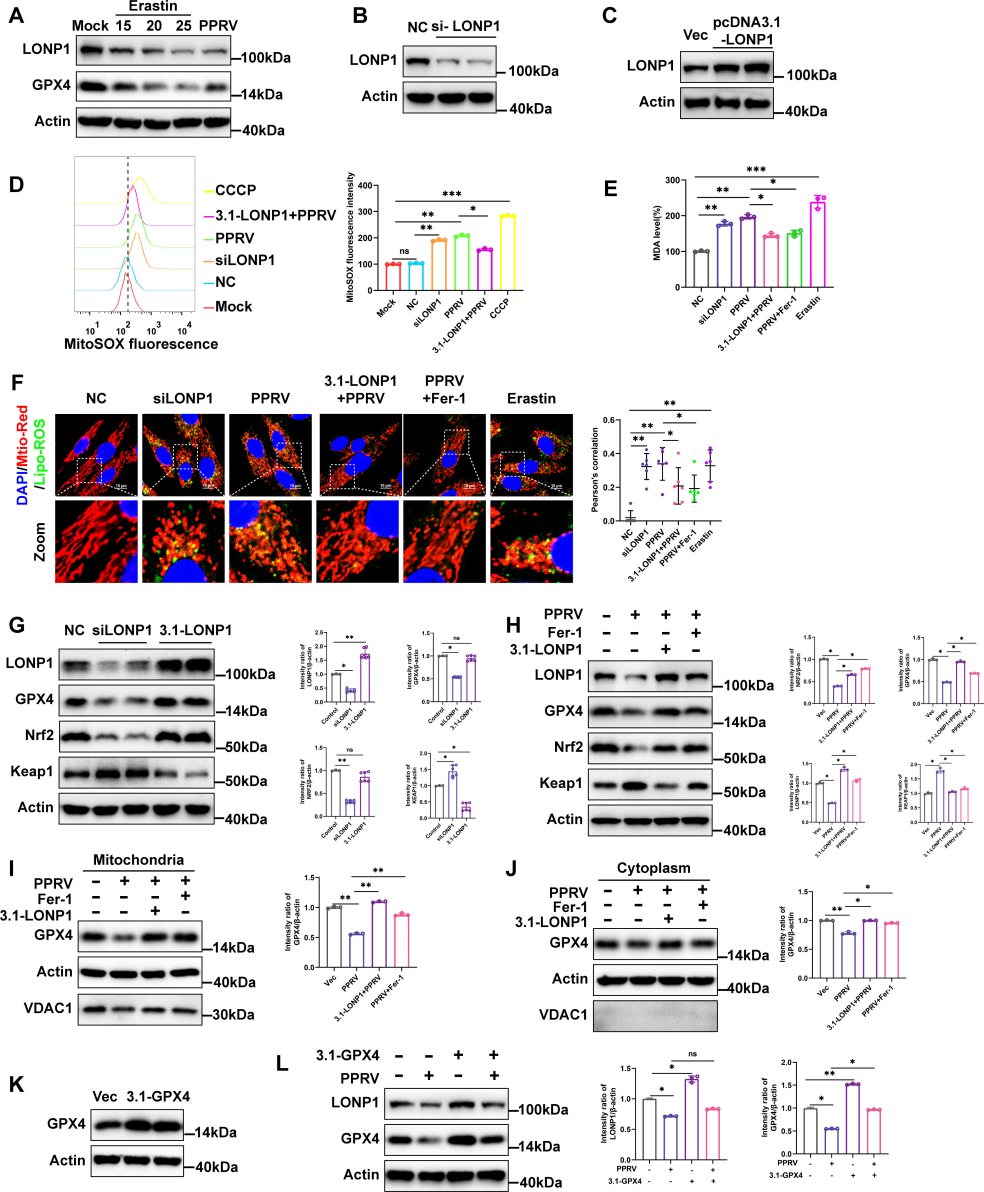
PPRV regulated the GPX4-Nrf2-Keap1 pathway by downregulating LONP1. (**A**) EECs were treated with Erastin (15, 20, or 25 µM) or infection with PPRV (MOI  = 5) for 48 h, Western blotting analysis of LONP1 and GPX4 and β-actin/Actin expression. Western blotting analysis of LONP1 expression in EECs transfected with siLONP1 or siNC for 48 h (**B**) and EECs were transfected with pcDNA3.1-LONP1 plasmids or pcDNA3.1 plasmids (**C**). (**D**) MitoSOX levels of control (Mock or NC), transfected with siLONP1 and PPRV infected with or without transfected with pcDNA3.1-LONP1 plasmid cells were detected by MitoSOX staining and analyzed by flow cytometry. Then, the mean MitoSOX fluorescence intensity of each cell was quantified. (**E**) Detection of MDA concentration in cell lysates. (**F**) Fluorescence analysis of the mitochondrial lipid peroxidation levels of control (NC), transfected with siLONP1, PPRV-infected cells, and PPRV infection treated with Fer-1 (20 µM) or transfected with pcDNA3.1-LONP1 plasmids and Erastin (20 µM) treatment cells stained for Liperfluo probe, Mito-Tracker, and Hoechst 33258. Scale bar = 10 µm. Colocalization analysis of immunofluorescence images using the colocalization plugin, which calculates Pearson’s correlation coefficient. (**G and H**) Western blotting and quantification of representative protein levels of LONP1, GPX4, Nrf2, Keap1, and β-actin/Actin (loading control) of EECs with knockdown and overexpression of LONP1 (**G**), control cells, PPRV-infected cells, and PPRV infection treated with Fer-1 (20 µM) or transfected with pcDNA3.1-LONP1 plasmid cells (**H**). (**I and J**) Western blotting analysis of GPX4 and VDAC1 proteins in mitochondrial fractions (**I**) and cytoplasmic fractions (**J**) from EECs PPRV infected and PPRV infection treated with Fer-1 (20 µM) or transfected with pcDNA3.1-LONP1 plasmid cells. (**K**) Western blotting analysis of GPX4 expression in EECs transfected with pcDNA3.1-GPX4 or pcDNA3.1 plasmids for 48 h. (**L**) Western blotting analysis of LONP1, GPX4, and β-actin/Actin protein levels in mock-infected and PPRV-infected cells with or without the transfection of pcDNA3.1-GPX4 or pcDNA3.1 plasmids at 48 h. The data are given as means ± SD from three independent experiments. *P* values were calculated using Student’s *t* test. An asterisk indicates a comparison with the indicated control. *, *P* < 0.05; **, *P* < 0.01; ***, *P* < 0.001; ns, not significant.

PPRV-induced ferroptosis shows the disturbances in mitochondrial morphology and function. Mitochondrial Lon protease 1 (LONP1) is one of the main multi-function enzymes regulating mitochondrial function and cytological stability (23). Our results revealed that PPRV infection downregulated protein (Fig. 4A and B) and mRNA (Fig. 4C and D) levels of LONP1 in a dose- and time- dependent manner. Of note, Erastin treatment reduced the expression levels of LONP1, which may be attributed to the severely damaged mitochondria (Fig. 5A). To evaluate the role of LONP1 in PPRV-induced ferroptosis, we transfected cells with siRNA targeting LONP1 (siLONP1), or pcDNA3.1-LONP1, or their respective controls, followed by PPRV infection. Western blotting analysis showed that transfection of siLONP1 effectively inhibited LONP1 compared to control cells (Fig. 5B), while the pcDNA3.1-LONP1 plasmid significantly overexpressed LONP1 (Fig. 5C). It seems that LONP1 knockdown increased mtSOX levels (Fig. 5D) and accumulation of lipid peroxidation levels in mitochondria (Fig. 5F), as well as MDA levels (Fig. 5E) compared to the transfected cells with siNC cells. In contrast, LONP1 overexpression decreased mtSOX levels (Fig. 5D), the accumulation of lipid peroxidation levels in mitochondria (Fig. 5F), and MDA levels (Fig. 5E) in PPRV-infected cells compared to cells only infected with PPRV. We used CCCP as a positive control, a known inducer of mitophagy (37). These results indicate that LONP1 could inhibit PPRV-induced accumulation of lipid peroxidation in mitochondria and reverse PPRV-induced ferroptosis in EECs. To further explore the details of LONP1 reverse PPRV-induced ferroptosis, we focused on the antioxidative genes implicated in negatively regulating ferroptosis. Our data showed that knockdown of LONP1 leads to decreased GPX4 and Nrf2 expression and increased Keap1 expression in EECs (Fig. 5G). The expression of GPX4 and Nrf2 did not change significantly, and the expression of Keap1 decreased in cells transfected with the 3.1-LONP1 plasmid (Fig. 5G). Importantly, overexpression of LONP1 or Fer-1 treatment rescued the decreased Nrf2 and GPX4 expression, as well as increased Keap1 expression in PPRV-infected cells compared to uninfected control cells (Fig. 5H). Furthermore, overexpression of LONP1 or Fer-1 treatment reversed the decreased GPX4 expression in both mitochondrial and cytoplasmic fractions in PPRV-infected cells (Fig. 5I and J). No detectable expression of VDAC1 was found in the purified cytoplasmic fractions from both PPRV-infected and uninfected cells, confirming the absence of mitochondrial contamination in cytoplasmic fractions (Fig. 5J). We also transfected cells with pcDNA3.1-GPX4, followed by PPRV infection. Western blotting analysis showed that transfection of pcDNA3.1-GPX4 plasmid significantly overexpressed GPX4 (Fig. 5K). Importantly, overexpression of GPX4 did not reverse the decrease of LONP1 protein level in PPRV infection (Fig. 5L). Together, we conclude that PPRV-induced LONP1 downregulation inhibits the activation of the Nrf2-Keap1 signaling pathway, leading to a decrease in GPX4 expression and promotion of ferroptosis.

### PPRV-induced ferritinophagy initiates ferroptosis

**Fig 6:**
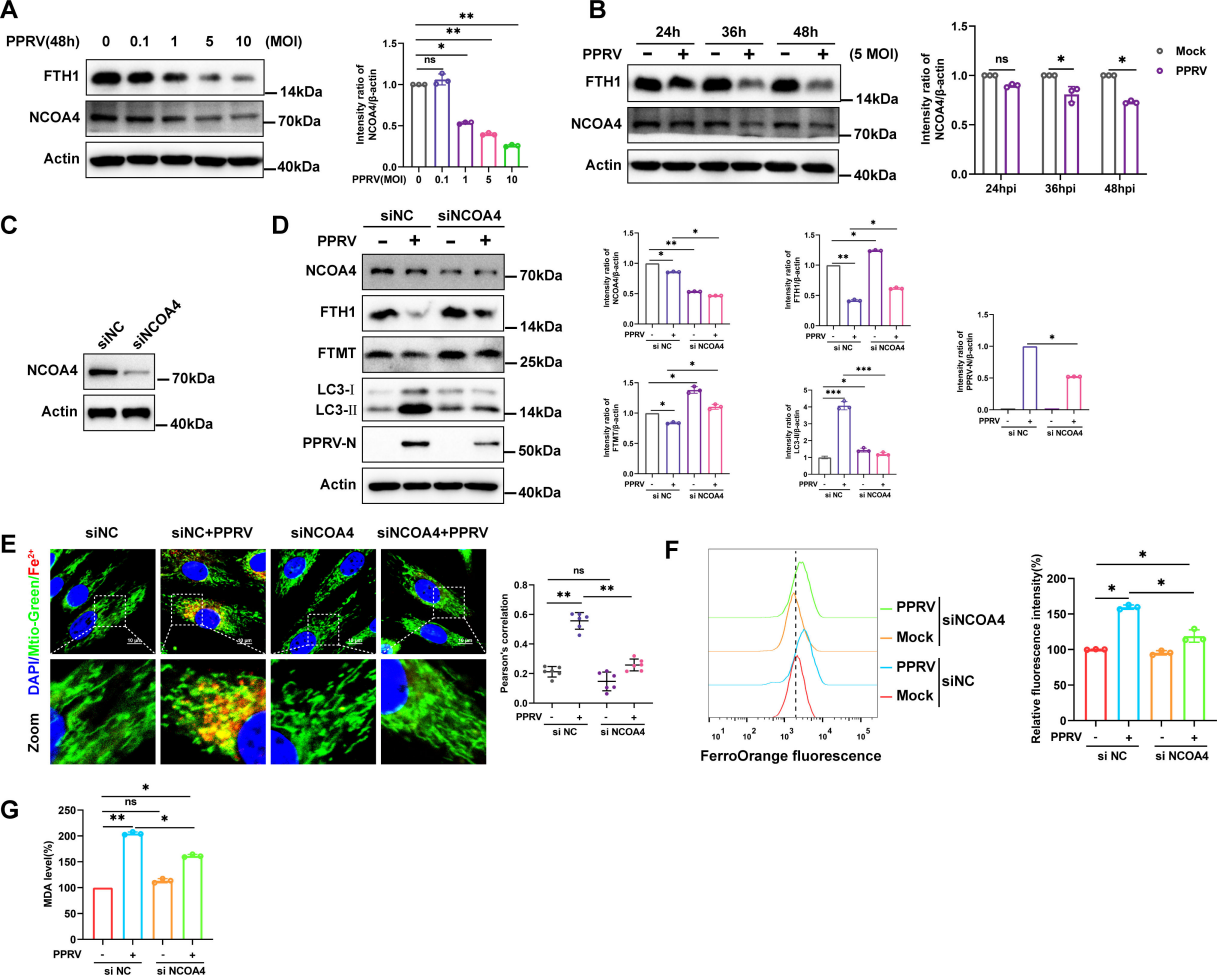
PPRV-induced ferritinophagy initiates ferroptosis. (**A and B**) FTH1, NCOA4, and β-actin/Actin (loading control) protein levels were determined via western blotting. (**A**) EECs were either mock treated or infected with PPRV Nigeria 75/1 at an MOI of 0.1, 1, 5, and 10. Whole-cell extracts were prepared from mock-infected and PPRV-infected cells at 48 h. (**B**) Cells were either mock treated or infected by PPRV at an MOI of 5 for 24, 36, and 48 h. (**C**) Western blotting analysis of NCOA4 expression in EECs transfected with siNCOA4 or siNC for 48 h. (D to G) EECs were mock-infected and infected with PPRV (MOI = 5) with or without the transfection of siNCOA4 or siNC at 48 h. (**D**) NCOA4, FTH1, FTMT, LC3, β-actin/Actin, and PPRV-N protein levels were determined via Western blotting and quantification. (**E**) Fluorescence analysis of the intracellular ferrous iron levels in mitochondria, the FerroOrange probe (red), the Mito-Tracker probe (green). Scale bar = 10 µm. (**F**) Flow cytometry analysis of intracellular ferrous iron levels. (**G**) Detection of MDA concentration in cell lysates. The data are given as means ± SD from three independent experiments. *P* values were calculated using Student’s *t* test. An asterisk indicates a comparison with the indicated control. *, *P* < 0.05; **, *P* < 0.01; ***, *P* < 0.001; ns, not significant.

Iron storage proteins, including FTH and FTMT, have a principal role in modulating ferroptosis through their ferroxidase activity (40). Iron release from ferritins is regulated by a process known as ferritinophagy (41). NCOA4 is a critical mediator for ferritinophagy activation (41). Western blotting analysis revealed that PPRV suppresses the expression of NCOA4 in EECs in a dose- and time-dependent manner (Fig. 6A and B), confirming that PPRV activates ferritinophagy. Correspondingly, decreased FTH1 in response to PPRV infection was also detected (Fig. 6A and B). To determine whether PPRV-induced ferritinophagy initiates ferroptosis, we used siRNA targeting NCOA4 (siNCOA4) to knock down the protein expression of NCOA4 (Fig. 6C). EECs were transfected with siNCOA4 or non-targeting control siRNA (siNC) for 48 h, followed by PPRV infection. Western blotting analysis showed that knockdown of NCOA4 increased the expression of FTH1 and FTMT, both in the presence and absence of PPRV (Fig. 6D), coinciding with the decreased Fe^2+^ accumulation in the cytoplasm and mitochondria, as observed by fluorescence staining analysis (Fig. 6E). Similar results were obtained by flow cytometry analysis (Fig. 6F). Accordingly, levels of MDA decreased after NCOA4 knockdown in PPRV-infected cells compared to siNC-transfected cells (Fig. 6G). Together, our findings suggest that PPRV-induced NCOA4-mediated ferritinophagy contributes to the accumulation of ferrous iron, initiating ferroptosis in EECs.

### PPRV-induced ferroptosis stimulates proinflammatory cytokine expression and inhibits type I IFN expression

**Fig 7:**
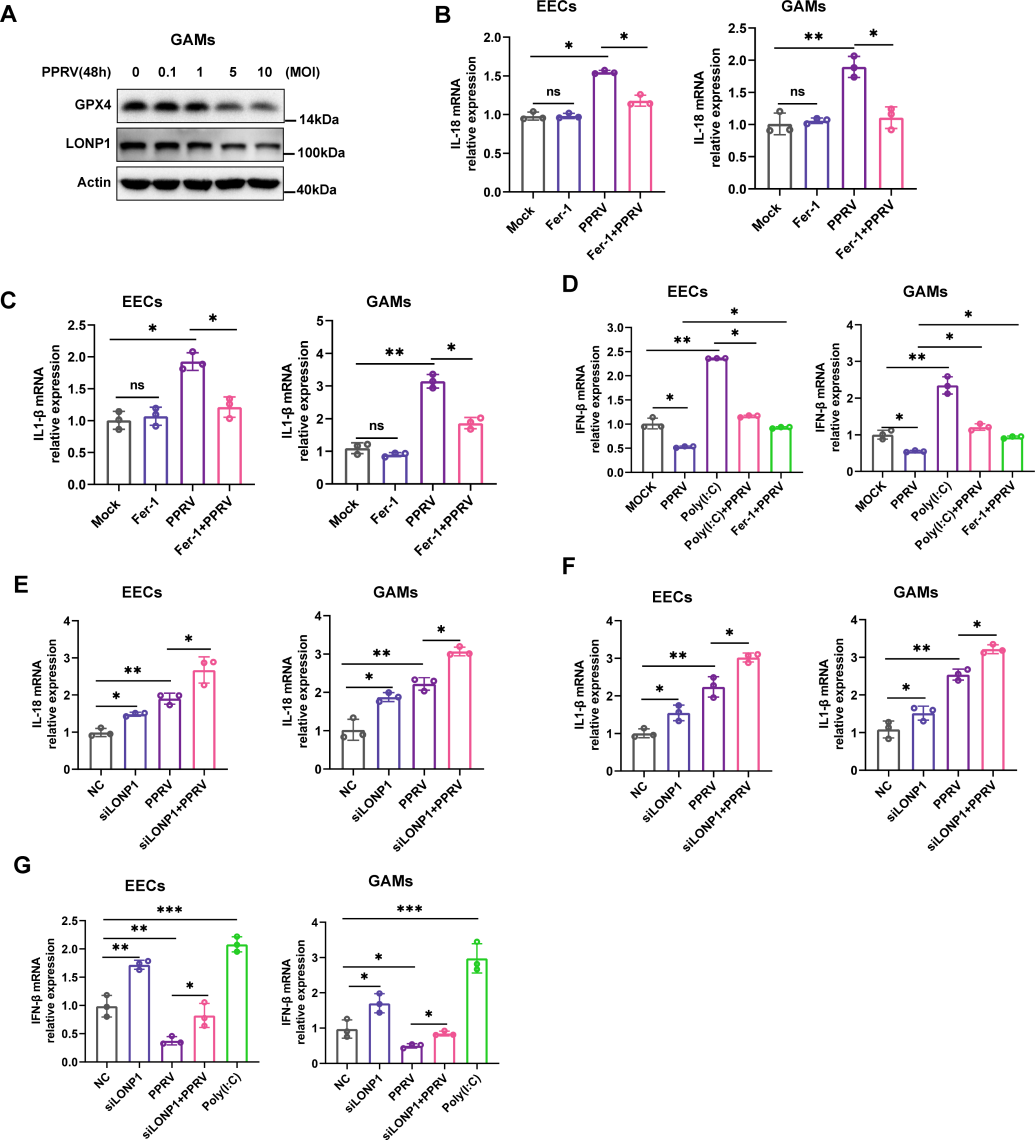
PPRV-induced ferroptosis stimulates proinflammatory cytokine expression and inhibits type I IFN expression. GAMs were either mock treated or infected with PPRV Nigeria 75/1 at an MOI of 0.1, 1, 5, or 10 for 48 h. GPX4, LONP1, and β-actin/Actin (loading control) protein levels were determined via western blotting (A). Real-time quantitative PCR analysis of IL-18 (**B and E**), IL-1β (**C and F**), and IFN-β (**D and G**) genes in PPRV (MOI = 5) at 48 h. (B to D) EECs were prepared from mock-infected, PPRV-infected cells with or without Fer-1 at 48 h. (E to F) EECs were mock-infected and infected with PPRV (MOI = 5) with or without the transfection of siLONP1 or siNC at 48 h. EECs were stimulated with poly(I·C) as the positive control (**D and G**). The data are given as means ± SD from three independent experiments. *P* values were calculated using Student’s *t* test. An asterisk indicates a comparison with the indicated control. *, *P* < 0.05; **, *P* < 0.01; ***, *P* < 0.001; ns, not significant.

Mitochondria play a pivotal role in inflammatory response (42), as well as in the regulation of many RCD processes, and ferroptosis is no exception (42). Studies have shown that the inflammatory responses of cells caused by pathogens are related to ferroptosis (43–45). Here, we investigated whether PPRV-induced ferroptosis plays a role in inflammatory responses during PPRV infection. Western blotting analysis revealed that PPRV infection suppressed the expression of GPX4 and LONP1 in goat alveolar macrophages (GAMs )in a dose -dependent manner (Fig. 7A). The results showed that PPRV also induces ferroptosis in GAMs. We measured the mRNA levels of proinflammatory genes, including interleukin-18 (IL-18), IL-1β, and IFN beta (IFN-β) in PPRV-infected EECs and GAMs in the presence or absence of Fer-1. Our data showed that PPRV infection significantly increased the expression levels of IL-18 (Fig. 7B) and IL-1β (Fig. 7C) and decreased the expression levels of IFN-β (Fig. 7D) compared to mock-infected cells. However, treatment with Fer-1 rescued the increased expression of IL-18 and IL-1β (Fig. 7B and C) as well as the reduced expression of IFN-β (Fig. 7D) in PPRV-infected cells. Poly(I·C)-treated cells were used as a positive control to stimulate IFN-β expression (4). Furthermore, this study explored the role of LONP1 in inflammatory responses in PPRV-infected EECs and GAMs. Knockdown of LONP1 enhanced the expression of IL-18, IL-1β, and IFN-β in the absence or presence of PPRV infection compared to cells transfected with control siRNA (Fig. 7E through G). Thus, PPRV-induced ferroptosis stimulates the expression of proinflammatory cytokines and suppresses type I IFN expression.

### PPRV H, N, and F proteins are involved in ferroptosis induction

**Fig 8:**
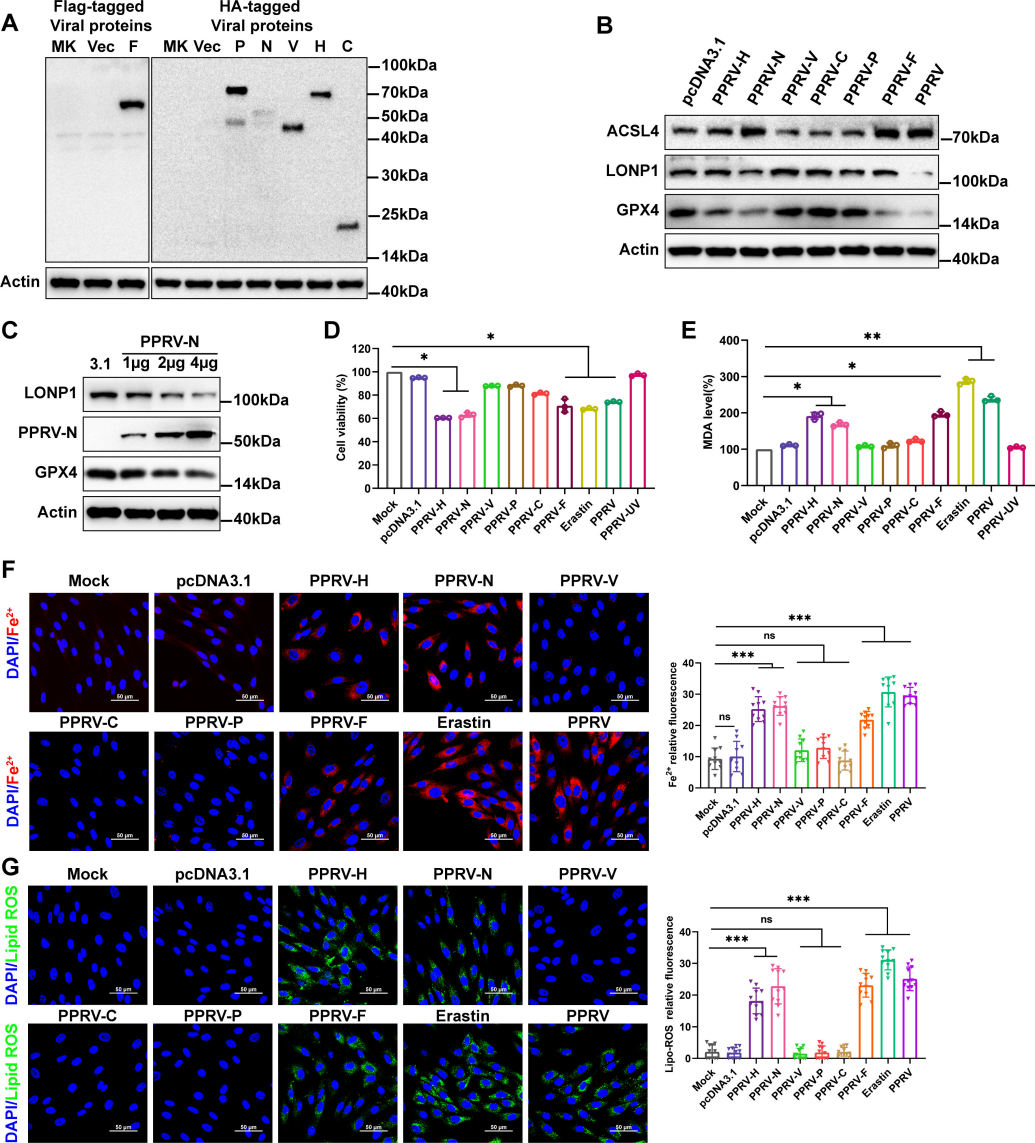
PPRV H, N, and F proteins are involved in ferroptosis induction. EECs were transfected with plasmids expressing various HA- or Flag-tagged viral proteins for 48 h and then subjected to western blotting with antibody against HA or Flag for the analysis of the expression of PPRV P, N, H, V, C, and F proteins in EECs (**A**). (**B**) Western blotting analysis of the protein levels of representative protein levels of ACSL4, LONP1, GPX4, and β-actin /Actin (loading control) of EECs with overexpression of PPRV H, N, V, C, P and F proteins, control cells, and PPRV-infected cells. (**C**) Western blotting analysis of LONP1 and GPX4 expression in EECs transfected with different doses of PPRV-N plasmids for 48 h. (**D**) The relative levels of cell viability were assayed by using cell CCK8. (**E**) Detection of MDA concentration in cell lysates. Representative images of EECs stained with FerroOrange (**F**) and with Liperfluo (**G**), treatment with Erastin and PPRV-infected cells as the positive control. Cell nucleus was counterstained with Hoechst 33258. Scale bars = 50 µm. The fluorescence intensity was quantified by ImageJ software. The data are given as means ± SD from three independent experiments. *P* values were calculated using Student’s *t* test. An asterisk indicates a comparison with the indicated control. *, *P* < 0.05; **, *P* < 0.01; ***, *P* < 0.001; ns, not significant.

To investigate the role of viral proteins that may be responsible for ferroptosis, EECs were transfected with plasmids expressing hemagglutinin (HA)- or Flag-tagged viral proteins for 48 h. Western blotting with antibodies against HA or Flag confirmed the expression of PPRV P, N, V, H, C, and F proteins in EECs (Fig. 8A). Western blotting analysis revealed that transfection with PPRV-H, N, or F plasmids suppressed the expression levels of GPX4 and increased ACSL4 expression levels in EECs compared to cells transfected with pCDNA3.1 or uninfected cells (Fig. 8B). Additionally, transfection with different doses of PPRV-N plasmids resulted in a dose-dependent decrease in the protein levels of GPX4 and LONP1 compared to cells transfected with pCDNA3.1 (Fig. 8C), further confirming the central role of PPRV-N protein in inducing ferroptosis. CCK8 analysis revealed decreased cell viability in cells expressing PPRV H, N, or F proteins, as well as in PPRV-infected and Erastin-treated cells compared to cells transfected with pCDNA3.1 or uninfected cells (Fig. 8D), accompanied by decreased MDA levels (Fig. 8E). Next, we measured the intracellular levels of Fe^2+^ and lipid peroxidation by staining with a Fe^2+^ and lipid peroxidation probe. Our data demonstrate that the H, N, and F proteins of PPRV significantly induce the accumulation of Fe^2+^ (Fig. 8F) and lipid peroxidation (Fig. 8G) compared with respective control groups. Taken together, these results indicate that the PPRV H, N, and F proteins may play key roles in inducing ferroptosis during PPRV infection.

## DISCUSSION

The diverse roles of mitochondria in ferroptosis have recently been explored, with mitochondria either promoting or suppressing ferroptosis under pathological conditions (14, 46). A high level of ROS, starvation due to decomposition of mitochondrial glutamine, and a high concentration of iron ions induce ferroptosis (17, 47, 48). Conversely, intact and normally functioning mitochondria resist ferroptosis (49). However, the interplay between mitochondria and the ferroptosis pathway during viral infection remains poorly understood. In this study, we provide evidence that mitochondria play a central role in ferroptosis induced by PPRV. Importantly, alterations of LONP1 regulation in response to PPRV infection cause mitochondrial dysfunction and subsequent induction of ferroptosis. Concurrently, PPRV-induced ferroptosis is tightly associated with inflammatory responses and enhanced virus replication levels.

Virus infections have been shown to trigger cell death via various mechanisms, depending on the viral species (29, 50, 51). Nevertheless, elucidating the causes and effects can be difficult. Ferroptosis is a regulated cell death pathway that heavily depends on iron-mediated lipid free radical formation and accumulation (12). During infection, the immune responses fortify their defense by withholding iron from pathogens (52, 53). However, various viral species have been found to interrupt iron uptake and the antioxidant response system, while others utilize iron transporter proteins as receptors (17). In the present study, PPRV infection and over-expression of H, N, and F proteins reduced the viability of EEC and concurrently induced characteristics of ferroptosis, including iron overload and lipid peroxidation. It should be noted that although PPRV and indicated viral proteins (H, N, and F) exhibit different properties when inducing ferroptosis, a low degree and similar levels of cell death were detected. Iron accumulation and lipid peroxidation may be considered as intermediate events, but they are not the final executors of ferroptosis (54). Not all damage caused by lipid peroxidation results in ferroptotic cell death in response to various stresses (55, 56). In addition, PPRV-N, PPRV-H, and PPRV-F proteins may participate in other pathways, such as autophagy, apoptosis, and immune responses during PPRV infection (5, 57–59). Moreover, inhibition of ferroptosis by Fer-1 abrogated ferroptosis caused by PPRV, as evidenced by the findings that lipid peroxidation decreased, and the levels of GPX4, FTH1, and FTMT were rescued. Furthermore, our findings indicate that PPRV-induced ferroptosis enhances viral replication levels. One possible explanation is that an increase in intracellular ROS levels can result in lipid peroxidation and cell death, which is beneficial for the release of virions and systemic viral spread (60). Additionally, increased ROS levels are closely linked to virus-induced metabolic reprogramming, which has been shown to support the replication of various viruses (47).

Ferroptosis results from the accumulation of cellular ROS, which subsequently assaults the bio-membrane system, leading to lipid peroxidation. Lipid ROS/peroxides, rather than cytosolic ROS, are the key drivers of ferroptosis (14). However, where and how lipid ROS generation during ferroptosis upon virus infection have not been defined. Mitochondria likely play a central role in ferroptosis, as they are the primary source of cellular ROS (14). Here, we monitored lipid ROS accumulation in PPRV-infected cells. Indeed, upon PPRV infection, increased levels of lipid ROS accumulation were observed mainly in mitochondria. We examined the subcellular localization of the lipid peroxidation probe by confocal imaging and found that, in PPRV-infected cells and Erastin-treated cells, the oxidized probe appeared to be distributed significantly colocalized with mitochondria rather than the cytoplasm. Therefore, our findings provide compelling evidence that mitochondria are indeed a crucial player in ferroptosis induced by PPRV.

The mitochondrial protease LONP1 has initially been identified as an enzyme involved in the degradation of damaged or misfolded proteins (24). However, a series of recent findings highlight the essential role that LONP1 plays in maintaining mitochondrial homeostasis and function, particularly in oxidative stress (61, 62). Several lines of evidence indicate that LONP1 acts as an antioxidant by degrading oxidized proteins in the mitochondrial matrix and limiting oxidative damage under hypoxic conditions (23, 63). Here, we observed downregulation of LONP1 in PPRV-infected cells, which was accompanied by decreased expression of GPX4 in the mitochondria and intracellular. GPX4 is the most critical ferroptosis defense gene that encodes cytosolic, mitochondrial, and nucleolar isoforms (38, 64). One of the key initial signals proposed to trigger ferroptosis is the inhibition of GPX4. Although the potential role of cytosolic GPX4 activity in various viruses-induced ferroptosis has been demonstrated (17, 32, 65, 66), it remains largely unknown whether mitochondrial GPX4 is an important component of ferroptosis during viral infections. Here, we observed that a higher abundance of GPX4 in mitochondria decreased more than that in the cytoplasm of PPRV-infected cells. Additionally, overexpression of LONP1 significantly rescued the decreased expression of GPX4 in mitochondria, indicating the crucial role of LONP1 in ferroptosis induction during PPRV infection. In addition, overexpression of GPX4 did not reverse the decrease of LONP1 protein level in PPRV infection. Notably, Erastin treatment reduced the expression levels of LONP1, which warrants further exploration. The antioxidant transcription factor Nrf2 is a key negative player in ferroptosis (67). Our data also demonstrate that the Nrf2/Keap1 pathway may be involved in the downregulation of GPX4 caused by decreased LONP1 induced by PPRV.

Ferroptosis results from the accumulation of cellular ROS that exceeds the cellular redox capacity. LONP1 also plays an important role in counteracting ROS production and maintaining mitochondrial homeostasis. The increased LONP1 levels can enhance resistance to external oxidative stress. Here, more ROS accumulated in mitochondria was observed in PPRV-infected cells. Overexpression of LONP1 counteracts ROS production during PPRV infection, suggesting the role of LONP1 in PPRV infection. However, the question of how decreased expression of LONP1 influences other mitochondrial functions and virus replication during PPRV infection remains an open issue.

Iron storage is equally important and can help prevent oxidative stress in cells caused by an excess of redox-active free iron. In this context, three iron storage proteins are known: FTH, FTL, and FTMT (40, 68). Both FTH and FTMT carry ferroxidase activity, enabling the storage of ferric hydroxides (Fe^3+^) rather than reactive ferrous iron (Fe^2+^) (40, 68). Iron release from ferritins is regulated by ferritinophagy, a process mediated by NCOA4; NCOA4 directly binds to FTH and transfers the complex to the autolysosome for degradation (32, 36). FTMT shows high sequence homology to FTH, which implies similar functionality (69). Recent studies have shown that FTMT inhibits oxidative stress-dependent ferroptosis by suppressing NCOA4-mediated ferritinophagy (70). Here, we demonstrate that the decreased expression of FTH and FTMT in PPRV-infected cells is linked to endogenous NCOA4 expression. When NCOA4 was knocked down using the siRNA approach, there was a considerable enhancement in the protein expression of FTH and FTMT. This observation of decreased endogenous NCOA4 through genetic manipulation or PPRV infection inducing a change in FTMT expression has not been previously reported. In addition to confirming FTH as an established target of NCOA4, we now provide evidence that NCOA4 also regulates FTMT during virus infection.

In summary, our results have established a novel link between mitochondria-mediated ferroptosis and inflammatory responses in response to PPRV infection. To the best of our knowledge, this study is the first to demonstrate the relationship between LONP1-mediated dysfunctional mitochondria, and the consequent induction of ferroptosis is involved in PPRV-induced pathogenesis, providing a promising therapeutic strategy to treat this important infectious disease with a worldwide distribution.

## MATERIALS AND METHODS

### Cell culture and virus

Caprine EECs were immortalized by transfection with human telomerase reverse transcriptase, and we have previously confirmed that the secretory function of these cells is consistent with that of primary EECs (71, 72). EECs were maintained in Dulbecco’s modified Eagle medium/F-12 Ham’s medium (DMEM/F12; Hyclone) medium containing 10% fetal bovine serum (FBS) (Gibco) and 1% penicillin-streptomycin at 37°C with 5% CO2. The PPRV attenuated strain Nigeria 75/1 was obtained in our laboratory on Vero cells. Viral load was conformed to cytopathic effect (CPE) and was quantified by assay of the 50% tissue culture infective dose (TCID50). The multiplicity of infection (MOI) was confirmed according to the viral titers of the respective cell lines. UV inactivation of PPRV was performed by irradiation with UV light for 1 h at room temperature.

### Antibodies and reagents

Anti-PPRV-N monoclonal antibody was provided by the China Institute of Veterinary Drug Control (Beijing, China). Antibodies to Keap1, NCOA4, and FTMT were purchased from Immunoway Biotechnology. Antibodies to GPX4, FTH1, Nrf2, ACSL4, and SLC7A11 were purchased from Abcom. Antibodies to Flag, HA, and LONP1 were purchased from Proteintech Group. LC3B, anti-mouse IgG, and anti-rabbit IgG, PE-conjugated goat anti-rabbit IgG (H + L), and fluorescein isothiocyanate-conjugated goat anti-rabbit IgG (H + L) were purchased from Sigma-Aldrich. Liperfluo and FerroOrange were purchased from Dojindo. MitoTracker Red/Green, MitoSox-Red, and JC-1 were purchased from Beyotime. Ferrostatin-1 and Erastin were purchased from Topscience (Hoechst 33258 [Solarbio]).

### Viral infection and cell treatment

During virus infection, EECs were seeded into 12-well cell culture plates at a density of 1  ×  10^5^ cells/ml and were inoculated with or without PPRV (Nigeria 75/1). After adsorption for 1.5 h, infected cells were maintained in DMEM/F12 medium (containing 2% FBS). The mRNA and protein levels of related factors were then determined by quantitative PCR (qPCR) and Western blotting, respectively. For the ferroptosis induction and inhibition experiments, EECs were pre-treated with Erastin (20 µM) and Ferrostatin-1 (Fer-1) (20 µM) for 6 h prior to viral infection. Viral adsorption was performed at 37°C for 1.5 h. The cells were then incubated in fresh medium containing Erastin (20 µM) and Fer-1 (20 µM) until the harvesting of the cells or the culture medium. Moreover, the same amount of dimethyl sulfoxide was added to the control group.

### RNA interference

SiRNAs targeting NCOA4 (target site: GGTCTCTCAAGACCATTCA), LONP1 (target site: GACCCUGAGCAGAAUGCAA), and GPX4 (target site: GGAGUAAUGCAGAGAUCAA) were designed and synthesized by Tsingke, Inc. (Beijing, China). SiRNAs were then used for silencing the target genes as described previously (3). The siRNAs were transfected into the cells using the transfection reagent Tubrofect (Thermo Fisher) according to the manufacturer’s instructions.

### Transmission electron microscopy

Semithin sections of cells were prepared and examined under a transmission electron microscope as described previously (5). EEC cells were mock-infected or infected with PPRV (MOI = 5) and in 6 cm dishes. The cells were then washed three times with phosphate-buffered saline (PBS) and collected by centrifugation at 1,000 × *g* for 5 min, prefixed with 2.5% glutaraldehyde, then postfixed in 1% osmium tetroxide, dehydrated in series acetone, infiltrated in Epox 812 for a longer time, and embedded. The semithin sections were stained with methylene blue, and ultrathin sections were cut with a diamond knife, stained with uranyl acetate and lead citrate. Sections were examined with a Hitachi HT-7700 transmission electron microscope (Hitachi, Tokyo, Japan).

### Western blotting analysis

Protein homogenates from the cells were extracted as previously described (5). Cells were lysed for 20 min on ice-cold lysis buffer (Roche). The lysates were centrifuged at 12,000 × *g* for 20 min at 4℃ to obtain a clear lysate. The protein content of each sample was determined using the BCA Protein Assay Kit (Thermo Scientific). Then, equal amounts of protein were separated on a 12% SDS-polyacrylamide gel and transferred to PVDF membranes (Merck-Millipore). Membranes were probed overnight at 4℃ with primary antibodies. The bands were visualized using horseradish peroxidase-conjugated goat anti-mouse IgG (1:5,000) or goat anti-rabbit IgG (1:5,000) prior to the ECL protocol (Merck Millipore). Signal was visualized using Konica SRX 101A developer (Konica Minolta Medical Imaging, Japan), and the Quantity One software (Bio-Rad, Mississauga, ON, Canada) was used for densitometrical analysis.

### Ferrous iron detection

The FerroOrange probe was used for flow cytometry and fluorescence imaging of intracellular Fe^2+^ (32). For the flow cytometry analysis, cells were trypsinized and resuspended with FerroOrange working solution (1 µM) and incubated at 37℃ for 30 min. and then assessed using a BD FACS Aria III High Speed Cell Sorter (BD Biosciences, San Diego, CA, USA), followed by analysis with FlowJo software, version 10 (Tree Star, Ashland, OR, USA). For fluorescence imaging of intracellular Fe^2+^, FerroOrange (1 µM) dispersed in serum-free medium was added to the cells, followed by incubation for 30 min at 37℃. Then, the cells were treated with Mito-Tracker Green (2 µM) at 37℃ for 30 min. The cells were then fixed with 4% paraformaldehyde for 45 min. Finally, the cells were treated with a Hoechst 33258 solution for 5 min. Cells were photographed under a confocal microscope (A1R; Nikon, Japan). The fluorescence intensity was analyzed using ImageJ software.

### MMP detection

MMP was measured using a JC-1 probe. The JC-1 probe was used for flow cytometry and fluorescence imaging of MMP. EECs were harvested with trypsin, then suspended in PBS, and immediately stained with JC-1, according to the manufacturer’s instructions. Then, the samples were analyzed with a BD FACS Aria III High Speed Cell Sorter (BD 602Biosciences, San Diego, CA, USA), followed by analysis with FlowJo software, version 10 (Tree Star, Ashland, OR, USA). For fluorescence imaging of MMP, after various treatments of each group, EECs were immediately stained with JC-1 according to the manufacturer’s instructions. Cells were photographed under a confocal microscope (A1R; Nikon, Japan). The fluorescence intensity of each treatment group for both aggregates (red) and monomeric forms (green) of JC-1 was analyzed using ImageJ software (NIH).

### Lipid peroxidation measurement

The levels of mitochondrial lipid peroxidation and intracellular lipid peroxidation were determined by using Liperfluo probe for flow cytometry and fluorescence imaging (31). For the flow cytometry analysis, cells were resuspended with Liperfluo working solution (1 µM), then incubated at 37℃ for 30 min, and then assessed using a BD FACS Aria III High-Speed Cell Sorter (BD Biosciences, San Diego, CA, USA), followed by analysis with FlowJo software, version 10 (Tree Star, Ashland, OR, USA). For fluorescence imaging of mitochondrial lipid peroxidation, cells were stained with Liperfluo working solution (1 µM) for 30 min at 37℃. Then, the cells were treated with Mito-Tracker Red CMXRos (1 µM) at 37℃ for 30 min. The cells were then fixed with 4% paraformaldehyde for 45 min. Finally, the cells were treated with a Hoechst 33258 solution for 5 min. Cells were photographed under a confocal microscope (A1R; Nikon, Japan). The fluorescence intensity was analyzed using ImageJ software.

### MDA assay

The detection of MDA concentration in cell lysates was performed strictly according to the manufacturer’s instructions (Beyotime, S0131S) (32). MDA is the major product of lipid peroxidation (33). In this assay, lipid peroxidation is determined by the reaction of MDA with thiobarbituric acid to form a colorimetric product (532 nm), which is directly proportional to the MDA concentration. The absorbance at 532 nm was measured using a plate reader (Bio-Rad).

### Real-time quantitative PCR analysis

Real-time quantitative PCR assay was performed as previously described (4). Total RNA was extracted from EECs using Trizol reagent (Invitrogen). RNA was then reversed using Superscript III (Invitrogen). Real-time quantitative PCR assay was carried out using an ABI 7500 System (Applied Biosystems, Warrington, UK). The PCR cycling conditions were 30 s at 94℃ followed by 40 cycles of 5 s at 94℃ and 30 s at 60℃. Real-time quantitative PCR assay of cytokine, PPRV-N, LONP1, and GAPDH mRNAs was carried out and calculated using the 2^−ΔΔCT^ method. PPRV-N, LONP1, IFN-β, IL-18, IL-1β, and GAPDH primers used in this study include the following:

PPRV-N, forward, 5′-GTGTCGGAGTCGAGTTGG-3′; PPRV-N, reverse, 5′-TTTGGCTTCCTCTGCTGT3′.

LONP1, forward, 5′-CACCGGAGGGTTCACATCAA-3′; LONP1, reverse, 5′-TCTGCCTCTTTCTTGCTCCG-3′.

IFN-β, forward, 5′-CTGCGGTGTGTCAGAAACTT-3′; IFN-β, reverse, 5′-CTTCCGGAACTGCTGTGCTT3′.

IL-18, forward, 5′-TGGCTGCAGAACCAGTAGAA3′; IL-18, reverse, 5′-TCTGATTCCAGGTCGCCATT-3′.

IL-1β, forward, 5′-CTCCAGCCAACCTTCATTGC-3′; IL-1β, reverse, 5′-GTTGGGTGCAGCTCTTCATCT3′.

GAPDH, forward, 5′-CTGCCCGTTCGACAGATAGC-3′; GAPDH, reverse, 5′-GCGGCCGAATCCGTTCA-3′.

### Mitochondria isolation

The mitochondrial fractions were isolated using the Cell Mitochondria Isolation Kit (Beyotime) according to the manufacturer’s instructions. The cells were digested and resuspended with cold PBS and centrifuged at 600 × *g* for 5 min at 4℃. The treated cells were resuspended and incubated in 1- to 2-mL ice-cold mitochondrial lysis buffer for 15 min. Subsequently, cell suspension was transferred to a glass homogenizer and homogenized for about 10–30 times. Then the cell homogenate was centrifuged at 600 × *g*, 4℃ for 10 min. The supernatant was collected and centrifuged at 11,000 × *g*, 4℃ for 10 min to isolate the mitochondrial fraction (pellet) by removing the cytoplasmic fraction (supernatant). Then the mitochondrial fraction was subjected to perform Western blotting analysis.

### Statistical analysis

All values are expressed as the arithmetic means of triplicates ± standard deviation (SD) from three independent experiments. Student’s *t*-test between two groups and one-way analysis of variance across multiple groups, followed by Tukey’s multiple comparison test using GraphPad Prism 6.0 software (GraphPad Software Inc., San Diego, CA, USA). *P < 0.05; **P < 0.01; ***, *P* < 0.001; ns, non-significant.

## Data Availability

The data supporting the findings of this study are available within the article.
